# Is Physical Activity Good or Bad for the Female Pelvic Floor? A Narrative Review

**DOI:** 10.1007/s40279-019-01243-1

**Published:** 2019-12-09

**Authors:** Kari Bø, Ingrid Elisabeth Nygaard

**Affiliations:** 1 Department of Sports Medicine, Norwegian School of Sport Sciences, PB 4014, Ullevål Stadion, 0806 Oslo, Norway; 2 Akershus University Hospital, Lørenskog, Norway; 3 Division of Female Pelvic Medicine and Reconstructive Surgery, Department of Obstetrics and Gynecology, University of Utah School of Medicine, Salt Lake City, USA

## Abstract

More women participate in sports than ever before and the proportion of women athletes at the Olympic Games is nearly 50%. The pelvic floor in women may be the only area of the body where the positive effect of physical activity has been questioned. The aim of this narrative review is to present two widely held opposing hypotheses on the effect of general exercise on the pelvic floor and to discuss the evidence for each. Hypothesis 1: by strengthening the pelvic floor muscles (PFM) and decreasing the levator hiatus, exercise decreases the risk of urinary incontinence, anal incontinence and pelvic organ prolapse, but negatively affects the ease and safety of childbirth. Hypothesis 2: by overloading and stretching the PFM, exercise not only increases the risk of these disorders, but also makes labor and childbirth easier, as the PFM do not obstruct the exit of the fetus. Key findings of this review endorse aspects of both hypotheses. Exercising women generally have similar or stronger PFM strength and larger levator ani muscles than non-exercising women, but this does not seem to have a greater risk of obstructed labor or childbirth. Additionally, women that specifically train their PFM while pregnant are not more likely to have outcomes associated with obstructed labor. Mild-to-moderate physical activity, such as walking, decreases the risk of urinary incontinence but female athletes are about three times more likely to have urinary incontinence compared to controls. There is some evidence that strenuous exercise may cause and worsen pelvic organ prolapse, but data are inconsistent. Both intra-abdominal pressure associated with exercise and PFM strength vary between activities and between women; thus the threshold for optimal or negative effects on the pelvic floor almost certainly differs from person to person. Our review highlights many knowledge gaps that need to be understood to understand the full effects of strenuous and non-strenuous activities on pelvic floor health.

## Key Points

**Table:** 

Exercising women have three times the risk of experiencing urinary incontinence.
Exercising women have larger cross-sectional area of the pelvic floor muscles but wider levator hiatus.
General exercise and pelvic floor muscle training during pregnancy have no negative effect on length of labor or mode of delivery.
Knowledge gaps prohibit firm conclusions about the role of strenuous physical activity in the incidence of pelvic floor disorders and highlight the need for further high-quality research.

## Introduction

Regular physical activity, that is, “any bodily movement produced by the skeletal muscles that results in a substantial increase over the resting energy expenditure” [1], is an important and modifiable health factor for all age groups, and there is evidence that “exercise is medicine” for a wide range of diseases and conditions [2]. In contrast to physical activity, which can be performed in varying domains, exercise training is a form of physical activity “usually performed on a repeated basis over an extended period of time with a specific external objective such as improvement of fitness, physical performance or health” [1]. The 2018 Physical Activity Guidelines Advisory Committee Report concluded that health benefits of regular physical activity include reduced risk of excessive weight gain, improved cognitive function, reduced risk of dementia and reduced risk of cancers in various sites [3]. In addition, physical activity reduces the risk of progression of some chronic conditions, such as osteoarthritis, hypertension, and type 2 diabetes. Hence, staying physically active throughout the lifespan is of great importance for health and well being.

 This message is clearly heard by women, as more and more participate in sports than ever before and the proportion of women athletes at the most recent Olympic Games was nearly 50%. Women and men differ in key areas of anatomy and physiology relevant to sports training, but perhaps of greatest difference is the often over-looked pelvic floor.

Indeed, the pelvic floor in women may be the only area of the body where the positive effect of physical activity has been questioned. The pelvic floor consists of muscles and connective tissues (ligaments and fascia) that need to work together to form a structural support for the pelvic organs to prevent urinary leakage or protrusion of the pelvic organs. Pelvic floor dysfunction may lead to common conditions such as urinary incontinence (UI), anal incontinence (AI) and pelvic organ prolapse (POP) [4]. Given that these conditions affect between one in three to one in four women [5, 6], understanding whether physical exercise might predispose to, or prevent, dysfunction of the pelvic floor, and thus these conditions, is important. Additionally, the conditions themselves, especially UI, may cause women to stop exercising or be one of many barriers to continuing lifelong regular physical activity [7, 8]. As a consequence, women and society bear the cost of inactivity and UI, both substantial [9, 10].

Known risk factors for pelvic floor disorders are pregnancy and vaginal childbirth, older age and obesity [11]. Strenuous work or exercise has also been widely debated as a possible risk factor. Indeed, the very definition of stress urinary incontinence [SUI], “complaint of involuntary loss of urine during effort or physical exertion, or during sneezing or coughing” [12], highlights the fact that leakage occurs during physical activity. Adequate function of the pelvic floor including the pelvic floor muscles (PFM), connective tissue and nervous system, is crucial in counteracting the increases in intra-abdominal pressure (IAP) and ground reaction forces that occur during physical activity, and well-functioning PFM may compensate for weak connective tissue.

In 2004, Bø described two possible and opposing hypotheses on the effect of physical activity on the pelvic floor [13]:

1.General exercise training strengthens the pelvic floor. The theories behind this hypothesis are that the impacts that occur during physical activity may stretch and fatigue the PFM, leading to a training effect, and/or that impacts during exercise could lead to a co-contraction of the PFM, creating an acute indirect training effect. This may reduce the levator hiatus area by causing hypertrophy and shortening of the surrounding muscles, thereby lifting the pelvic floor and the internal organs into a higher pelvic location. Theoretically, such morphological changes could reduce the risk of UI, AI and POP. On the other hand, it is also theoretically possible that these changes could negatively impact labor and childbirth by making it more difficult for the fetus to descend with pushing.

2.General exercise training overloads, stretches and weakens the pelvic floor. This hypothesis is based on the fact that physical activity increases IAP, and if the pelvic floor muscles are not able to co-contract quickly or strongly enough to counteract this increased pressure or withstand the ground reaction forces, the levator hiatus could become wider, stretching and weakening the muscles. According to this theory, overload of the PFM may increase the risk of UI, AI and POP, but on the other hand, should also result in easier childbirth.

The aim of this narrative literature review is to describe and discuss the evidence supporting or refuting these two hypotheses, including how exercise influences PFM strength, muscle fatigue, pelvic floor morphology, pelvic floor disorders, and labor and birth variables.

## Methodological Considerations

We based our paper on previous review articles in this area and articles from the reference lists of these articles [13–20] and on an updated search on February 2019. The search strategy in PubMed included the terms: (prevalence OR incidence) AND (“stress urinary incontinence” OR “urinary incontinence” OR “urine incontinence” OR “fecal incontinence” OR “faecal incontinence” OR “pelvic organ prolapse”) AND (athletes OR athlete OR exercisers OR sport [tw] OR sports [tw]; *N* = 66 and (exercise OR “physical activity”) AND (“intra-abdominal pressure” OR “intraabdominal pressure” OR “abdominal pressure”) AND “pelvic floor”; *N* = 52. Of these 118 studies, 23 and 35, respectively, did not address the areas of interest, and 20 studies were represented in both searches, leaving 40 published studies from PubMed for review, in addition to the review manuscripts. We also included seven manuscripts not found in our search to incorporate papers we were aware are not included in the PubMed database.

## Findings

### How Do Specific Exercises Influence Intra-Abdominal Pressure?

IAP changes throughout the course of the day, increasing with position changes, movement, breathing, and abdominal wall contraction. Two exercise modalities may increase IAP to a greater extent than others and thus possibly affect the pelvic floor: strenuous strength training, such as weight and power lifting, and high-impact activities, such as jumping and running. Strength training and weight lifting are characterized by short-duration bursts of impact with possible high increases in IAP, but low ground reaction forces [15]. High-impact activities are associated with a high number of impacts from both ground reaction forces and (probably smaller) increases of IAP. Continent women automatically pre- or co-contract the PFM before and during impact activities and are not aware of this automatic function [21]. Women with SUI can learn to voluntarily pre- and co-contract the PFM when coughing [22]. This would in theory also be possible during some strength-training exercises or heavy lifting of short duration, but this has not yet been investigated. However, in long-lasting activities with repeated impacts close together in time, such as running or high-impact aerobics, it would be impossible for women to perform voluntary PFM contractions for each step or movement.

To have an understanding of the forces the pelvic floor must withstand, it is useful to consider these forces during propagation. Hay [23] estimated maximum vertical ground reaction forces during different activities and reported those during running to be 3–4 times the body weight, jumping, 5–12 times, landing from a front somersault, 9 times, landing from double back somersault, 14 times, and long-jump, 16 times. Since then, many authors have investigated vertical ground reaction forces and confirmed substantial forces that occur during running, walking and jumping. While out of the scope of the current review, it is instructive to summarize one example. Seegmiller et al. studied ground reaction forces in drop landings, which produce a characteristic two-peak curve, in ten competitive gymnasts [24]. Scaling the force data to N/kg to account for differences in body mass among subjects, they reported that the first peak vertical force magnitudes ranged from 9.5 N/kg at 30-cm height to 32.8 N/kg at 90-cm height, while the second much greater peak vertical force magnitudes ranged from 27.1 at 30-cm height to 56.0 N/kg at 90 cm. While most women are unlikely to experience these types of loads on the pelvic floor, artistic gymnasts may do so during the landing phases of many of their routines.

Measuring pressures in the bladder, rectum, or upper vagina provides a closer approximation of the forces experienced by the pelvic floor, although discounting acceleration forces. James was one of the first to measure bladder pressures during physical activity, using an air-filled balloon in the bladder [25]. Recorded pressures during coughing, jumping and running and bending over to the floor were 125, 90 and 20 cmH_2_O, respectively.

Subsequent studies demonstrate clearly that maximal IAP values have a very wide range amongst women doing the same standardized activity [26]. In addition, maximal IAPs vary across studies for the same activity, in part related to the instrumentation with which IAP is measured and how maximal IAP is constructed, as well as to differences between populations [27, 28]. Breathing pattern is generally not standardized, which can also influence IAP [29]. IAPs across studies also vary because both total (that is, maximal IAP from atmospheric pressure baseline) and net (difference between maximal IAP and a baseline value, usually standing) are used to operationalize IAP but usually not specified in reports.

Examples of IAPs during dynamic activities and abdominal training are summarized in Tables 1 and 2. In an interesting study challenging the widely held belief that pelvic floor “safe” exercises generate lower IAPs than corresponding conventional exercises, no differences in IAPs were found between the recommended and discouraged versions of half the exercises, including ball rotations, lunges, core, push-ups and squats [30]. Others have also pointed out that activities generally restricted after surgery may generate lower IAPs than unrestricted activities. For example, mean maximal IAP was greater with standing up from a chair than it was for abdominal crunches, climbing stairs, sit-ups and many lifting activities [31]. Similarly, lifting 20 lbs generated less IAP than standing up from a chair [32]. Coughing generates higher IAP than most exercises (Table 1).

**Table 1:** Examples of mean maximal intra-abdominal pressures generated during dynamic activities^a^

Study	*N*	Walking	Jumping	Running	Coughing
Weir et al. [31] (2006)	30^b^	Treadmill, 3.3 mph 79.0 (48–190) cmH_2_O	Jumping jacks 127 (59–190) cmH_2_O		
O’Dell and Morse [106] (2007)	12			Jogging in place 54 (27–76) cmH_2_O	98 (50–131) cmH_2_O
Kruger et al. [107] (2013)	12	Treadmill, 6 km/h 38 (N/A) cmH_2_O	Star jumps 53 (NA) cmH_2_O	On treadmill 7 km/h 45 (NA) cmH_2_O	73 (N/A) cmH_2_O
Shaw et al. [108] (2014)	57	Treadmill, 4.8 km/h 25 (15–37) cmH_2_O		On treadmill 8–9.7 km/h 67 (32–99) cmH_2_O	91 (38–200) cmH_2_O
Yamasato et al. [32] (2014)	147 with POP/SUI				80 (14–150) cmH_2_O
Coleman et al. [109] (2014)	46	Running track 43 (14–79) cmH_2_O slow pace to 62 (40–110) cmH_2_O fast pace			
Simpson et al. [110] (2016)	30 with SUI/POP				78 (14–84) cmH_2_O
DeGennaro et al. [111] (2017)	25	Treadmill, 3.4 mph at 14% grade 69 (46–102) cmH_2_O	Jumping jacks 124 (78–189) cmH_2_O		

*POP* pelvic organ prolapse, *SUI* stress urinary incontinence, *N/A* not applicable (no range provided)

^a^All pressures were measured using vaginal catheters/sensors with the exception of Weir et al. [31], who measured pressure using a rectal catheter

^b^Unless otherwise specified, participants did not report incontinence

**Table 2:** Examples of mean maximal intra-abdominal pressures generated during abdominal exercise and lifting^a^

Study	*N*	Abdominal exercise	Heavy lifting	Lighter lifting
Mouritsen et al. [112] (2007)	23^b^			Lift 5.0 kg 22.3 (N/A) cmH_2_O
O’Dell and Morse [106] (2007)	12		Lift 20.4 kg 71 (51–120) cmH_2_O	
Gerten et al. [113] (2008)	41		Lift 15.0 kg 82 (N/A) cmH_2_O	Lift 2.5 kg 48 (N/A) cmH_2_O
Shaw et al. [108] (2014)	57	Curl-up: 19 (7–82) cm H_2_O Full sit up: 60 (14–129) cm H_2_O	13.6 kg 35 (17–63) cmH_2_O 18.2 kg 48 (14–120) cmH_2_O	
Yamasato et al. [32] (2014)	147 with SUI/POP			Lift 4.5 kg 12 (2–38) cmH_2_O Lift 9 kg 19 (5–64) cmH_2_O
Coleman et al. [114] (2015)	16	Plank: 38 (23–60) cmH_2_O Roll-up on mat: 51 (33–76) cmH_2_O Roll-up on Pilates reformer: 50 (29–74) cmH_2_O		
Simpson et al. [110] (2016)	30 with SUI/POP	Curl-up: 50 (17–100) cmH_2_O		
DeGennaro et al. [111] (2017)	25	Curl-up: 27 (9–66) cmH_2_O Full sit up: 64 (28–133) cmH_2_O Plank: 49 (23–95) cmH_2_O		
Hsu et al. [115] (2017)	206 6–10 weeks postpartum		Lift 12.5 kg 54 (26–80) cmH_2_O	

*POP* pelvic organ prolapse, *SUI* stress urinary incontinence, *N/A* not applicable (no range provided)

^a^All pressures were measured using vaginal catheters/sensors with the exception of Gerten et al. [113], who measured pressure using a rectal catheter

^b^Unless otherwise specified, participants did not report incontinence

In a study using a novel intra-vaginal pressure sensor that simultaneously measures PFM contraction and IAP during a selection of supine exercises in 21 women’s health physiotherapists, the mean vaginal pressure during a PFM contraction increased 16.3 mmHg (SD 12.3) while the corresponding IAP increase was minimal [mean 3.4 mmHg (SD 2.2)] [33]. During an abdominal crunch, the IAP increased 8.3 mmHg (SD 7.3); simultaneously the pressure measured at level of the PFM also increased, indicating co-contraction, to half that of the PFM contraction [mean 8.2 mmHg (SD 8.0)].

### Does General Exercise Strengthen or Weaken the Pelvic Floor Muscles?

#### Does One Bout of Strenuous Exercise Fatigue the PFM?

We found only two studies measuring the acute effect of one bout of exercise. In a short-term experimental cross-over study of young nulliparous women with symptoms of SUI, there was a 17% reduction of maximum voluntary PFM contraction after a 90-min session that included strenuous high-impact endurance and strength training, but no change in vaginal resting pressure or muscular endurance [34]. In contrast, immediately after one bout of strenuous exercise in women that habitually performed CrossFit and one bout of non-strenuous exercise in recreational controls, there was no change in maximum voluntary PFM contraction, but there was a decrease in vaginal resting pressure in both groups, as well as slightly worse vaginal support [35].

We are aware of no prospective studies following women from onset of exercise over months or years of training to evaluate the effect of exercise training on PFM strength.

#### Do Exercisers have Stronger or Weaker PFM than Non-Exercisers?

Some studies have compared PFM strength between exercising women and controls. These studies provide mixed evidence for answering the question of whether exercisers have stronger or weaker PFM than non-exercisers.

##### Evidence in Support of Stronger PFM

Compared to 44 healthy women, 49 high-impact athletes had stronger PFM than these controls [36]. In a group of 41 women, PFM strength correlated with aerobic capacity and also with habitual physical activity measured by questionnaire [37]. Controlling for PFM training and other factors, primigravid women performing general exercise at 21 week gestation ≥ 3 times per week had stronger and more enduring PFM than non-exercisers [38].

##### Evidence in Support of Weaker PFM

Volley- and basketball players (*n* = 10 each) had significantly weaker PFM than non-exercising controls [39].

#### Evidence Suggesting No Difference in PFM Strength

In a study of 70 healthy women, PFM strength did not differ between those habitually engaged in CrossFit and controls [35]. Another study of 100 postmenopausal women reported no linear relation between physical activity and PFM strength after adjusting for other factors [40]. Similarly, there were no differences in PFM strength between 30 women with no clinical diagnosis of pelvic floor disorders who were Pilates practitioners and sedentary controls [41]. In a cross-sectional analysis of 203 primiparous women 1 year postpartum, there were no significant associations between PFM force and measures of strength and fitness, including grip strength, trunk flexor endurance duration, percent body fat, or self-reported physical activity [42]. Similarly, another cross-sectional study found no association between physical activity level, assessed by questionnaire, and vaginal resting pressure, PFM strength and endurance [43]. However, there was a weak positive association between physical activity level in continent women and a weak negative association in incontinent women [43].

#### How Does Exercise Affect PFM Activity?

Some researchers have used vaginal surface electromyography (EMG) to measure activity attributed to the PFM during various activities. In ten healthy women, vaginal surface EMG activity was higher during running at 11 km/h compared to 7 or 9 km/h [44]. In another study of 16 healthy women, EMG activity during jumps occurring while drop landing and mini-trampolining was above that of the PFM onset threshold and pre- and co-contraction activity increased significantly with jumping height and body weight force [45]. A review of 28 studies about PFM activity during impact activities, concluded that the timing of PFM activity in relation to the activity of other trunk muscles appears to be important in maintaining continence and that women with SUI have delayed PFM activity during impact activities [46].

### What is the Effect of Exercise on Pelvic Floor Morphology?

There were no differences in the size of the pelvis or urogenital hiatus on magnetic resonance imaging (MRI) in ten athletes versus ten age-matched controls, but the cross-sectional area of the levator ani muscle was 20% higher in the athletes [47]. Using 3D/4D translabial ultrasound, 24 nulliparous high-impact athletes had a larger levator hiatus area on Valsalva, greater levator ani muscle diameter, and greater bladder descent during Valsalva than 25 age- and body mass index (BMI)-matched controls [48]. These results were somewhat contradicted by findings that five incontinent football players had significantly greater levator muscle thickness at the midvagina on MRI than seven continent players [49]. Another study found no difference in vaginal support between 35 women habitually engaged in CrossFit and controls [35]. In primiparas, there was no difference in levator hiatus area at 21 weeks gestation between women who reported exercising for 30 or more minutes at least three times per week and non-exercisers, but at 37 weeks, exercisers had significantly larger levator hiatus area both at rest and during Valsalva [38]. The proportion of women that reported also doing PFM training three or more times per week was similar between groups, about 15%.

In a group of 90 women of whom 60% reported SUI and 25% were unable to contract the PFM, all displayed bladder-based depression on ultrasound when performing abdominal curls. Breathing pattern was not standardized. There were no differences between women with or without SUI, but parous women displayed significantly larger depression than nulliparous women [50].

### What is the Effect of Exercise on Urinary Incontinence?

We identified no randomized controlled trials (RCTs) evaluating the effect of physical activity or general exercise training on UI that did not also include pelvic floor muscle training (PFMT). Numerous cross-sectional and fewer cohort studies have evaluated the association between exercise and UI.

#### Less UI in Exercisers

Mild-to-moderate physical activity, largely represented by walking, appears to decrease the risk of UI. In cross-sectional analyses, current leisure activity is associated with lower odds of SUI; conversely the lack of exercise increases these odds [51–55]. Similarly, several prospective cohort studies, most notably the Nurses’ Health Study, concluded that greater levels of physical activity decreased the risk of developing new UI and also decreased the risk of persistent UI [56–58].

#### More UI in Exercisers

In a comparison of 213 middle-aged women with moderate/severe SUI and 213 aged-matched women with no/mild SUI, SUI odds increased slightly with overall lifetime activity, but was not associated with lifetime strenuous activity, assessed using the Lifetime Physical Activity Questionnaire [53]. A large body of the literature supports a high prevalence of UI in women participating in sports. Prevalence rates are not directly comparable between studies due to different instruments and definitions of UI and population differences. In previous reviews, the prevalence of reported UI during sports ranged from 28% in university varsity athletes to 80% in teenaged nulliparous trampolinists [14–20]. The prevalence is generally greater in high-impact athletes, such as trampolinists, gymnasts, volleyball players, and long-distance runners [15].

All studies [36, 59–65], except Bø and Borgen [66] and Dockter et al. [67] comparing UI prevalence between athletes and controls report significantly higher prevalence in athletes. For example, Fernandes et al. reported that 63% of 12–19 year old amateur soccer players demonstrated objective evidence of UI compared to 25% of similarly aged girls not participating in sports [62]. Systematic reviews have concluded that the odds of UI in athletes/exercising women may be 3.5 times that of controls [17, 18].

There are sparse data on the incidence of UI after initiating exercise or sports training. A prospective study found no difference in the prevalence of SUI before and after a 6-week program of summer military training completed by 116 young nulliparous women [68].

#### UI and PFM Strength in Exercisers

While exercising women at 37 weeks’ gestation had stronger PFM than non-exercising women, the UI prevalence was not different between groups, after adjusting for PFM training, age, pre-pregnancy body mass index, education and smoking [38]. An adjusted linear regression model showed that PFM strength, rather than being a regular exerciser, was associated with continence. In another study, athletes (gymnasts, distance runners and basketball players) had significant higher UI prevalence than sedentary women, despite also having greater PFM strength [36]. Somewhat counter-intuitively, incontinent athletes in a different study had stronger PFM than continent athletes [69].

#### Long-Term Effects of Strenuous Exercise on UI

In a study questioning U.S. Olympians, 20 years after they competed, there were no differences in UI prevalence between a high-impact group (former gymnasts and track and field) and a low-impact group (swimmers) [70]. Similarly, in a 15 year follow-up study, former Norwegian elite athletes, including those participating in high-impact sports, were not more likely to report UI later in life than controls [71]. However, UI early in life was strongly associated with UI at 15 year follow-up. In contrast, in a population of middle-aged women, the predicted probability of SUI rose linearly, though modestly, in those that recalled exceeding 7.5 h of strenuous activity/week during their teen years, even after adjusting for subsequent strenuous activity during ages 21–65 years [53].

### What is the Effect of Exercise on Anal Incontinence?

Few studies were found on exercise and AI. We identified no studies on weight/power lifters, in whom anecdotally AI may be expected, and no long-term studies.

#### Less AI Among Exercisers

We identified no studies reporting that women exercising have less AI.

#### More AI Among Exercisers

The reported prevalence of AI amongst athletes aged 18–40 years was 14.8% in 169 intensive sport athletes compared to 4.9% in 224 non-intensive women; for the majority, the reported anal incontinence was categorized as flatus [60]. In an Internet survey of 311 female triathletes, 28% reported anal incontinence [72].

#### Evidence Suggesting No Difference in AI Between Exercisers and Controls

A retrospective study of 40 female athletes and 80 matched controls found that no woman in either group reported fecal incontinence (a subset of anal incontinence, which includes incontinence with flatus or with stool) during pregnancy or several years after pregnancy [73].

### What is the Effect of Exercise on Pelvic Organ Prolapse?

There are fewer studies on the relationship between physical activity/exercise and POP than there are for UI.

#### Less POP in Exercisers

No studies were found reporting less POP in exercisers.

#### Evidence Suggesting No Difference in POP Between Exercisers and Controls

In 144 nulliparous women at the United States Military Academy, 50% had stage I or II POP, which represents mild pelvic floor support deficits, but former and current exercise did not increase odds of POP [74].

Brækken et al. compared 49 women with POP stage ≥ 2 with 49 women stages 0–1 and found that BMI, socioeconomic status, heavy occupational work, anal sphincter lacerations and PFM function were independently associated with POP, whereas joint mobility and low intensity physical activity were not [75]. In a cross-sectional analysis of middle-aged women, there were no associations between odds of POP and overall reported lifetime physical activity, lifetime leisure activity, or lifetime strenuous activity between 191 POP cases and 191 age-matched controls [76]. There was a marginally significant nonlinear relationship between teen strenuous activity and POP amongst women reporting > 21 h/week of teen strenuous activity.

#### More POP in Exercisers

We identified no studies concluding that POP prevalence is higher in exercising than non-exercising women. However, a prospective study of 116 nulliparous female soldiers before and after summer military training concluded that the subset that also did paratrooper training (*n* = 37) were significantly more likely to have stage II POP than those that did only basic training, as well as worsening of their initial pelvic floor support [74].

Compared to findings after overnight bedrest, immediately after 1 h of structured exercise, more severe POP was found on examination in a group of 54 women planning surgery for POP [77]. One bout of CrossFit exercise increased vaginal descent in another study [35]. In the Internet survey of female triathletes, 5% endorsed symptoms consistent with POP [72].

### What Factors Increase the Risk of UI in Exercising Women?

We identified few consistent factors. In 144 nulliparous, college varsity athletes, there were no significant associations between UI and amenorrhea, weight, hormonal therapy or duration of athletic activity [78]. In former Olympians, only current BMI, but not age, parity or Olympic sport, was associated with regular UI symptoms [70]. Another study of elite athletes found that SUI prevalence was greater in those with an eating disorder, but there were no differences in either menstrual function or BMI between elite athletes with and without SUI [71]. Incontinent trampoliners were older (16 vs 13 years old) and had been training longer and more frequently than those that were continent [79]. A study of 623 athletes from different sports also found that UI prevalence increased with age and long training hours [80].

Compared to continent group fitness instructors, those with UI were older, less likely to use oral contraceptives, and had taught group fitness classes longer [81]. In a population of track and field, basketball and indoor football players, those reporting UI had lower BMI than those that were continent, but there was no difference in age [82]. While 24% of triathletes in an Internet survey had at least one arm of the female athlete triad (disordered eating, menstrual irregularities, osteoporosis), this was not associated with pelvic floor disorder (PFD) symptoms [72]. Amongst 104 athletes from different sports, only years of training predicted increased risk of evidence of UI based on a pad test [83]. In another study of 50 female athletes, after multivariable adjustment, hours of training per day similarly increased UI odds [84].

### How Does Exercise Affect Labor and Birth?

#### More Difficult Labor and Birth

We identified no studies reporting that exercising women have more difficult labor and births than non-exercising women.

#### Easier Labor and Birth

A systematic review by the 2016 Evidence summary of the International Olympic Committee expert group meeting on exercise and pregnancy in recreational and elite athletes concluded that exercise did not increase the risk of induction, epidural, episiotomy or perineal tears, and forceps or vacuum delivery, but appeared to be associated with a shorter first stage of labor and lower risk of cesarean delivery [85]. Similarly, a recent systematic review and meta-analysis concluded that based on moderate quality evidence from 20 exercise-only RCTs conducted antenatally (*n* = 3819), the odds of instrumental delivery in women who exercised was lower than those who did not; there were no differences between groups in any other labor/delivery variables studied [86]. However, the conclusions were based on few studies (mostly retrospective and prospective observational studies) and the studies had low-to-moderate quality [85, 86].

In a prospective observational study following 274 primiparous women, the levator hiatus area during both rest and valsalva widened during pregnancy [87] and regular exercisers, defined as exercising ≥ 30 min ≥ 3 times/week at 37 weeks’ gestation had larger, not smaller, levator hiatus area than non-exercisers [88]. There were no differences in delivery outcomes between exercisers and non-exercisers.

In terms of exercise directed at the pelvic floor (pelvic floor muscle training), two observational studies did not find that antenatal PFM training made labor and birth more difficult [89, 90]. Furthermore, a systematic review including 12 RCTs or quasi RCTs (*n* = 2243) concluded that prenatal PFM training significantly shortened both the first and second stages of labor [91]. The training did not increase the risk of episiotomy, instrumental vaginal delivery or perineal lacerations. However, only three trials with high clinical heterogeneity contributed to the first stage of labor analysis and six trials with high clinical and moderate statistical heterogeneity were pooled in the second stage of labor analysis.

## Discussion

To summarize key findings of this review, exercising women may have similar or stronger PFM and larger levator ani muscles than non-exercising women, but this does not have a greater risk of obstructed labor or childbirth. Women that perform PFM training antenatally also are not more likely to have outcomes associated with obstructed labor (such as prolonged second stage of labor or cesarean delivery), but rather, appear to have shorter first and second stages of labor; this conclusion is limited by lower quality data. In terms of pelvic floor dysfunction, urinary incontinence is common amongst exercising women, with exercise increasing the odds 2.5–3 times and with greater prevalence rates associated with higher impact activities. Mild-to-moderate physical activity, such as walking, may decrease the risk of future UI. Scant research suggests that strenuous activity in younger women does not predispose to UI in later life, though a large volume of strenuous activity during the teen years might. The few studies available to assess the association between exercise and POP and AI are inconsistent in their conclusions. The rigor of these findings is limited by inconsistencies in the literature in defining each of the pelvic floor disorders and in methods of assessing and characterizing pelvic floor muscle strength, morphology, and intra-abdominal pressure.

In terms of IAP, variability amongst women is high. Some exercises thought to be associated with higher IAP are in fact not, and many generate lower IAPs than common daily activities. However, whether higher IAPs directly affect the pelvic floor is not known. In addition to the actual load on the pelvic floor, repetitions of loading and dynamic activities may impact the pelvic floor differently than the actual load.

Measuring PFM function during physical activity and specific exercises is very challenging. Results from studies attempting this are difficult to compare. Electromyography (EMG) may provide a means of assessing the kinematic aspects of PFM contraction, and not simply the pressures obtained, during exercise. Whether methods such as surface EMG are valid in assessing PFM activity is debated given input from other muscle groups during multitask activities such as running and jumping [92]. EMG is not a measure of muscle strength, but of activation of muscle fibers. Strength and activation are correlated, but measure different features. Vaginal pressure transducers designed to measure PFM strength may be impacted by IAP increases. Measurement devices to be kept inside the urethra, vagina or rectum during exercise may move, impacting results. Even in the laboratory setting, measuring PFM strength can be challenging. It is vital to ensure that women are indeed contracting the PFM correctly. We are not aware of commercially available instruments that measure increases in IAP and function of the PFM simultaneously, but new developments are being investigated [28, 33]. However, given the location of the PFM inside the pelvis, their close connection to other muscle groups and their inclusion in the abdominal canister that responds to all IAP and ground reaction forces, it may prove difficult to differentiate between opposing forces occurring during physical activity.

Studies investigating PFM strength and exercise are cross-sectional, and thus causality cannot be established. That one session of strenuous exercise results in acute PFM fatigue does not provide any evidence about whether such fatigue might cause stronger or weaker PFM in the long term [34]. The effect of IAP on pelvic floor function requires not only consideration of the PFM, but also of other co-existing factors. We suggest that there may be an individual threshold of IAP related to each individual’s harm/benefit ratio. Most women can tolerate huge increases in IAP without leaking. Hence, the connective tissue and the PFM are adequately counteracting this increased pressure and corresponding downward movement. In others, only small amounts of IAP may move the pelvic floor downwards [93], widen the levator hiatus [94] and decrease the maximal urethral closure pressure, causing leakage or descent.

Most of the literature about exercise and pelvic floor dysfunction is limited to UI with few studies about AI and POP, and is generalizable to recreational exercisers with scant data about strenuous exercisers and elite athletes. Most large epidemiological studies on physical activity and UI are cross-sectional. The inherent selection bias with this design, especially for a condition like UI, may be large, as women with UI tend to stop exercising. These study designs cannot ascertain whether women are exercising because they are dry or whether they are dry because they exercise. Many epidemiological studies do not control for obesity, which is important as obesity is a risk factor for UI and exercising women are more likely to be within normal weight distribution, thus reducing their risk of UI [11].

Although exercisers may have stronger PFM than non-exercisers, the PFM may still be too weak or too slow to counteract the IAP or ground reaction forces during strenuous activities [21]. It is reasonable for athletes to consider focused strength training of the PFM, particularly if they have symptoms of UI, given the grade A recommendation for PFM training as first line treatment for UI in the general female population [95]. Furthermore, pregnant continent women who exercise the PFM (primary prevention) are 62% less likely to experience UI in late pregnancy and have a 29% lower risk of UI 3–6 months postpartum [96]. In women with POP, supervised PFM training results in lift of the pelvic floor, a smaller levator hiatus area and PFM hypertrophy [97].

Whether such training effects are found in elite athletes remains to be investigated. Several small uncontrolled studies suggest that athletes and soldiers demonstrate improvements in symptoms or PFM strength after PFM training [98–100]. In a more recent RCT, in 32 volleyball players, UI was more likely to improve after PFM training than after written information only [101]. However, young, nulliparous women in general, and athletes in particular, have low level of knowledge about the pelvic floor and little knowledge about how to train the PFM [102].

Elite athletes are accustomed to regular training and are highly motivated for exercise. Adding 3 sets of 8–12 close to maximum PFM contractions, 3–4 times a week to their regular strength-training programs is feasible [101, 103]. Proper strength training of the PFM seems to be important as one RCT found that simply contracting the PFM during daily activities does not itself seem to improve PFM function and prevent UI [104]. While a minority of athletes performed a correct PFM contraction at their first assessment in one study, all learned proper technique after individual instruction by a physiotherapist [69]. We are not aware of studies investigating whether exercising women are more likely to adhere to PFM training.

Because most elite athletes are nulliparous, one does not expect damage to ligaments, fascia, muscle fibers or peripheral nerves. Therefore, the effect of PFM training in athletes might be equal to or better than that of other women. On the other hand, the increases in IAP and ground reaction forces that must be counteracted automatically by the athlete’s PFM are higher than that required in the general population. The pelvic floor, therefore, probably needs to be much stronger and respond more quickly to forces in elite athletes than in the lay public.

The prevalence of UI during sports is high. Given the numerous health benefits of physical activity, no one should be recommended to stop exercising, and this is certainly not an option for elite athletes. However, athletes should be provided with the same information and advice to train the PFM as the rest of the female population. Athletes do not talk about UI with their coaches or trainers [78, 81, 105]. Education directed at coaches has potential to improve UI in athletes.

## Conclusion

In conclusion, our review identified evidence to support both hypotheses about the effect of physical activity on the pelvic floor. However, data are scant, particularly regarding POP and AI, studies generally cross-sectional, confounders are often not considered, and there is substantial variability in case definitions and assessment methods. These limitations highlight the need for further high-quality research. To enable women to reap the benefits of physical activity without being affected by, or affecting, the pelvic floor, there is an urgent need for a body of research, summarized in Table 3. There is more support (though still scant) for the hypothesis that strenuous exercise may be a risk factor for PFD, than there is for regular exercise being of benefit for PFM function. For women with an optimally functioning pelvic floor, it is likely that physical activity has a positive training effect, through unconscious co-contraction of the PFM, while for those that do not, strenuous exercise may be deleterious. Athletes with an optimally functioning pelvic floor likely demonstrate a continuum of positive and negative responses to impact; e.g., a gymnast may have a pelvic floor that can withstand the forces generated from landing on the floor exercise mat, but may leak during landing on the beam. The threshold for optimal or negative effects on the pelvic floor almost certainly differs from person to person.

**Table 3:** Research priorities to understand the associations between physical activity, the pelvic floor, and pelvic floor disorders and the impact of pelvic floor disorders on sports performance and participation

**Understudied populations**
Elite athletes
Weight trainers
Pregnant athletes
**Understudied outcomes**
Anal incontinence
Pelvic organ prolapse
Labor and delivery: duration of first and second stages, instrumental delivery, cesarean delivery
**Under-represented research designs**
Prospective cohort studies
Randomized clinical trials
With untreated control groups
Large and generalizable samples
**Important research questions**
**Pelvic floor muscle training**
What is the effect of PFMT in recreational athletes on PFD symptoms?
What is the effect of PFMT in women exposed to high loads on the pelvic floor (such as high-impact athletes or weight lifters) on PFD symptoms?
Does the effect of PFMT in athletes on PFD symptoms differ according to type of sport (static versus dynamic)?
Other than PFMT, what treatment modalities may improve PFD symptoms in athletes?
Is PFMT cost effective in athletes?
What is the knowledge base of coaches and trainers about PFM and PFDs?
How can PFMT best be incorporated into athletes’ training regimens?
How does PFMT affect athletic performance?
**Prospective assessment of effect of exercise on the pelvic floor**
How does commencing heavy and strenuous exercise impact the PF?
What is the effect of general exercise training (excluding PFMT) on PFM strength and function?
What is the long-term impact of teen strenuous activity on PFM strength and function, and on PFDs?
What is the long-term impact of strenuous activity initiated after teenaged years on PFM strength and function and PFDs?
**Effect of exercise on PFD symptoms**
What effect does initiating a sports or exercise training program have on urinary incontinence incidence or resolution?
What are modifiable risk factors for PFDs in elite athletes and strenuous exercisers?
How does UI affect athletic performance?
What beliefs and social constructs do athletes place on UI?
What is the effect of strenuous exercise during pregnancy on subsequent PFD symptoms?
What are the prevalence rates of UI in teenaged athletes performing different types of sports?
**Effect of exercise on intra-abdominal pressure**
How does exercise training affect IAP during different types of activities?
How does IAP generated during single vs repetitive and static and vs dynamic activities affect PFM strength and function and PFD symptoms?
How does breathing pattern during exercise affect IAP?
**Assessment**
How can measuring PFM strength and function accurately during physical activity be improved upon?
How can kinematic aspects of PFM function best be assessed?
What other methods can be developed to measure the influence from physical activity on the pelvic floor?

*PFM* pelvic floor muscle, *PFMT* pelvic floor muscle training, *PFD* pelvic floor disorder, *PF* pelvic floor, *UI* urinary incontinence, *IAP* intra-abdominal pressure
